# The associations between mast cell infiltration, clinical features and molecular types of invasive breast cancer

**DOI:** 10.18632/oncotarget.13163

**Published:** 2016-11-07

**Authors:** Jianfeng Sang, Dandan Yi, Xiaoqiao Tang, Yifen Zhang, Tao Huang

**Affiliations:** ^1^ Department of General Surgery, Nanjing Drum Tower Hospital, Gulou District, Nanjing 210008, Jiangsu Province, China; ^2^ Department of General Surgery, Drum Tower Clinical Medical College of Nanjing Medical University, Gulou District, Nanjing 210008, Jiangsu Province, China; ^3^ Department of Pathology, Affiliated Hospital of Nanjing University of Chinese Medicine, Jiangsu Province Hospital of Traditional Chinese Medicine, Qinhuai District, Nanjing 210009, Jiangsu Province, China

**Keywords:** breast cancer, mast cell, estrogen receptor, progestogen receptor

## Abstract

Associations between mast cell infiltration and the clinical features and known molecular profile of breast cancer remain unclear. The distribution difference of mast cell was evaluated, in 219 patients with no special type of invasive carcinoma, using sorts of age, max diameter of cancer, histological type, lymph node metastasis as well as the expressions of estrogen receptor (ER), progestogen receptor (PR), human epidermal growth factor receptor 2 (HER-2) and nuclear protein Ki67. The mast cell density (MCD) in patients younger than 50 years old was significantly higher than that in patients with age ≥ 50. The MCD in ER or PR positive patients was significantly higher than MCD in ER or PR negative patients. The MCD in patients with Ki67 ≤ 14% was also significantly higher than MDC in patients with Ki67 > 14%. The MCD of patients with invasive ductal carcinoma was significantly higher than MCD of patients with invasive lobular carcinoma. No significant distribution difference of MCD was found to be associated with max diameter of cancer, lymph node metastasis and HER-2. Further analysis found that MDC was significantly higher in patients after neo-adjuvant chemotherapy. The distribution difference of mast cell widely exists in patients with distinct clinical features, the role of mast cell in breast cancer need further research with detailed and reasonable classification to clarify.

## INTRODUCTION

In the humoral immune process, plasma cells synthesize and secrete IgE antibody when meet alien antigen; the IgE could bind to the high-affinity IgE receptor (FcεRI) located in the mast cell membrane and will active mast cell to participate humoral immunity [1]. Activated mast cells gradually degranulate and release the bioactive substances, which recruit inflammatory cells and induces inflammation reaction [1]. Studies show that mast cells accumulated in a variety of malignant tumors such as melanoma tumor [2], Hodgkin's lymphoma [3], pancreatic cancer [4], prostate cancer [5], esophageal cancer [6] and chronic lymphocytic leukemia [7, 8] and associated with poor prognosis and distant metastasis [2-8]. These reports suggested that mast cells play a promoting role in the occurrence and development of malignant tumors [2-8]. The mechanism may be related to the inflammatory mediators and pro-angiogenic factors (e.g., stem cell factor, SCF) secreted by tumor cells. Mast cells highly expressed differentiation antigen 117 (CD117), which is a ligand for SCF [9, 10]. The interaction between SCF and CD117 might recruit mast cells in tumor tissue and make mast cells secret fibroblast growth factor-2 (FGF-2) and vascular endothelial growth factor (VEGF) and hence promote tumor growth [11]. In addition, mast cells secret various protease, especially tryptase, which can activate latent metalloproteinase, in turn caused extracellular matrix structure and composition changes, capillary formation and activation of vasoactive substances [12, 13]. On the contrary, studies also found that mast cells have a role in suppressing tumor development. In colorectal cancer, high mast cell infiltration was found in patients with less lymph node metastasis and distant metastasis [14]. In vitro experiments also found mast cells can inhibit the expression of c-myc in hepatocellular carcinoma, and displayed antitumor effects via release of tumor necrosis factor (TNF), interleukin prime (IL) and other substances [15]. Histamine secreted by mast cells can stimulate vascular endothelial cell to produce prostacyclin, which could induce necrosis of tumor cells and inhibit the metastasis of tumor cells [16]. Oldford et al observed that IL-6 secreted by mast cells can activate toll like receptor-2 (TLR-2) which can inhibit tumor in vivo and in vitro [17]. Maltby et al also found that trypsin and IL-5 secreted by mast cells can promote the recruitment and activation of eosinophils, thereby inhibit tumor growth [18].

Existing studies on the relationship between the mast cell infiltration and the prognosis of breast cancer are also inconsistent [19-21]. Some scholars believe that the higher the density of mast cells in the breast cancer tissue, the worse the prognosis. Kashiwase et al found that, mast cell density was higher in invasive breast cancer and in breast carcinoma with poor differentiation and poor prognosis than that in fibroadenoma and tubular carcinoma and papillary carcinoma with good prognosis [22]. Some other scholars hold opposing views, they believe that mast cell infiltration suggest the good prognosis of breast cancer. A study of 4,444 patients with invasive breast cancer showed that the infiltration of mast cells was an independent good prognostic indicator [23]. Study on breast cancer patients with axillary lymph node metastasis showed that the number of mast cells in the tumor tissues is less in patients with greater axillary lymph node metastasis [24]. The occurrence and development of any tumor is a systematic biological process with the participation of multiple factors. The above inconsistent results suggest further studies on the role of mast cell in breast cancer are needed.

As is known, estrogen receptor (ER), progestogen receptor (PR), human epidermal growth factor receptor 2 (HER-2) and nuclear protein Ki67 are the main known biomarkers to evaluate the prognosis of breast cancer [25-27]. ER and PR positive or negative can be used for preliminary evaluation of hormone therapy [27]. HER-2 overexpression indicates higher degree of malignancy, disease progression is faster, more likely to relapse and metastasis [27]. The percentage of Ki67 expression was closely related to the degree of differentiation, invasion, metastasis and prognosis of many tumors [26]. Even though, these markers, including the Luminal system, have limited contribution to clinical practice, more and more works are needed to improve current methods of diagnosis, treatment and prognosis prediction of breast cancer.

In this report, the infiltration of mast cells was studied in 219 patients with invasive breast cancer, the association between mast cells infiltration and expression of ER, PR, HER-2, Ki67 as well as Luminal system were evaluated.

## RESULTS

### Clinical characteristics of participants

Totally, 219 subjects with invasive breast cancer were assembled in this report, their clinical characteristics of patients without neo-adjuvant chemotherapy were summarized in the Table 1. The median age is 52.0 (with interquartile range: 46.0 to 59.0); 51.1%, 4.1%, 95.9% and 41.6% were subjects with left breast cancer, invasive lobular carcinoma, invasive ductal carcinoma and axillary lymph node metastasis, respectively (Table 1). The median of max diameter of cancer is 2.2 (with interquartile range:1.5 to 3.0) cm (Table 1); 67.1%, 65.3% and 23.7% subjects were ER, PR and HER-2 positive, respectively (Table 1). 30.0% (with interquartile range: 15.0% to 50.0%) breast cancer samples were Ki67 positive (Table 1). 17.8%, 43.4%, 24.2% and 14.6% patients were categorized as luminal A, luminal B, HER-2 overexpression and triple negative respectively (Table 1).

**Table 1:** Clinical characteristics of the study subjects (N = 219)

Age (year)	52.0 (46.0, 59.0)
Left breast cancer	112 (51.1%)
Right breast cancer	107 (48.9%)
Max diameter of cancer (cm)	2.2 (1.5, 3.0)
Invasive lobular carcinoma	9 (4.1%)
Invasive ductal carcinoma	210 (95.9%)
Axillary lymph node metastasis	91 (41.6%)
ER+	147 (67.1%)
PR+	143 (65.3%)
HER-2+	52 (23.7%)
Ki67	30.0% (15.0%, 50.0%)
Luminal A	39 (17.8%)
Luminal B	95 (43.4%)
HER-2 overexpression	53 (24.2%)
ER-, PR- and Her-2-	32 (14.6%)

Abbreviations: Continuous variables are presented as median (with interquartile range); categorical variables are presented as number (percentage). ER+, estrogen receptor positive; PR+, progesterone receptor positive; HER-2+, human epidermal growth factor receptor-2 positive; Ki67, Ki67 nuclear antigen.

### The distribution difference of mast cell sorted by age, max diameter of cancer, histological type and lymph node metastasis

**Figure 1:**
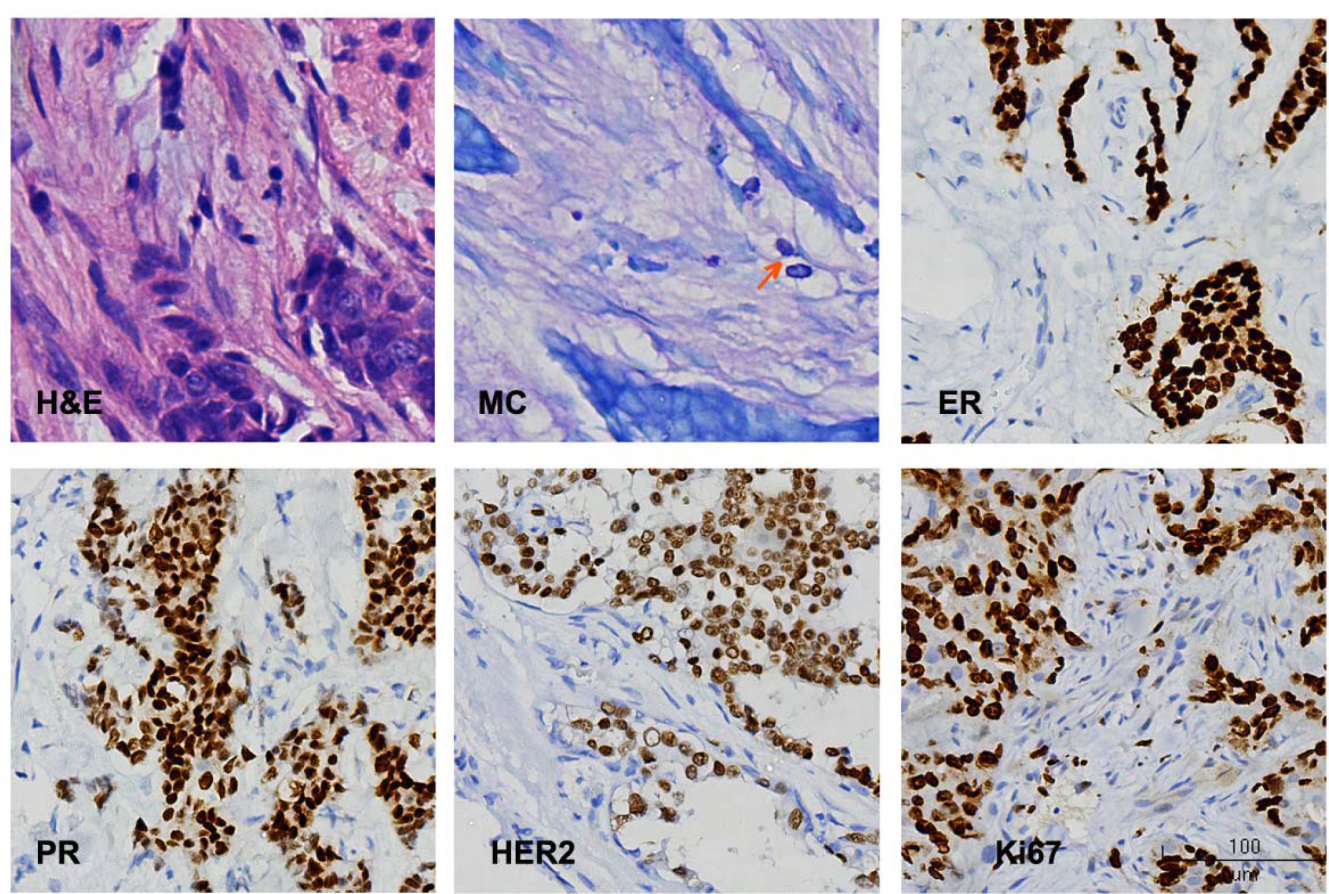
Representative histological staining H & E, hematoxylin and eosin staining; MC, mast cells, arrow indicates MC; ER, estrogen receptor; PR, progesterone receptor; HER-2, human epidermal growth factor receptor-2; Ki67, Ki67 nuclear antigen.

Histological examination as well as mast cell infiltration were evaluated by H&E and toluidine blue staining (Figure 1).

To study the distribution difference of mast cell between menopause patients and reproductive patients, the mast cell densities (MCD) of breast cancer samples from patients < 50 years and patients ≥ 50 years were counted. The average rank of MCD in patients < 50 years were significantly higher than that in patients ≥ 50 years (133.9 vs. 93.9, *P* < 0.001) (Table 2).

**Table 2:** The distribution difference of mast cell sorted by clinical and molecular factors

Variable	N	Average rank	Z	*P*
*Age, year*				
< 50	88	133.9	-4.589	***<0.001***
≥ 50	131	93.9		
*Max diameter of cancer (cm)*				
≤ 2.0	101	112.7	0.335 (χ^2^)	*0.846*
2.0 - 5.0	111	107.7		
> 5.0	7	108.4		
*Histological type*				
Invasive ductal carcinoma	210	107.9	-2.322	***0.020***
Invasive lobular carcinoma	9	157.9		
*Lymph node metastasis*				
Yes	128	108.7	-3.530	*0.724*
No	91	111.8		
*ER*				
Negative	72	91.4	-3.052	***0.002***
Positive	147	119.1		
*PR*				
Negative	76	95.3	-2.508	***0.012***
Positive	143	117.8		
*HER-2*				
Negative	167	111.9	-0.807	*0.420*
Positive	52	103.8		
*Ki67*				
≤ 14%	49	132.8	-2.861	***0.004***
> 14%	170	103.4		
*Molecular typing*				
Luminal A	39	133.8	13.423(χ^2^)	***0.004***
Luminal B	95	112.8		
HER-2 overexpression	53	105.9		
ER-, PR- and HER-2-	32	79.4		

Abbreviations: ER+, estrogen receptor positive; PR+, progesterone receptor positive; HER-2+, human epidermal growth factor receptor-2 positive; Ki67, Ki67 nuclear antigen; N, number of subjects in each group; Z, Z-value.

There were 210 and 9 patients were invasive ductal carcinoma and invasive lobular carcinoma, respectively, to learn if any distribution difference of MCD exists between the two histological types, MCDs were also calculated. The average rank of MCD in patients with invasive ductal carcinoma was significantly lower than that in patients with invasive lobular carcinoma (107.9 vs. 157.9, *P* = 0.020).

The distribution differences of MCD between patients with different max diameter of cancer and between patients with and without lymph node metastasis were also evaluated. Our data showed that max diameter of cancer and lymph node metastasis did not associate with MCD (Table 2).

### The distribution difference of mast cell sorted by known biomarkers

To learn whether MCD associated with known molecular biomarkers of breast cancer, the expressions of ER, PR, HER-2 and Ki67 in cancer samples of each patient were detected using immunohistochemical method (Figure 1). The average ranks of MCD in ER and PR positive cancers were significantly higher than these in ER and PR negative cancers (119.1 vs. 91.4, *P* = 0.002; 117.8 vs. 95.3, *P* = 0.012, respectively) (Table 2). The average rank of MCD in cancers with Ki67 ≤ 14% was significantly higher than that in cancers with Ki67 > 14% (132.8 vs. 103.4, *P* = 0.004) (Table 2). No significant difference of MCD was observed between HER-2 negative and positive patients (Table 2).

### The associations between MCD and molecular types

The average ranks of MCD in luminal A, luminal B, HER-2 overexpression and triple negative breast cancer patients were 133.8, 112.8, 105.8 and 79.4 respectively (Table 2). Kruskal-Wallis test showed significant difference exist among molecular types (X^2^ = 13.423, *P* = 0.004). Further Nemenyi's multiple comparison showed that the average ranks of MCD in luminal A and luminal B were significantly higher than these in triple negative breast cancer patients (P < 0.01 and = 0.04, respectively).

### The association between MCD and chemotherapy

To study the association between MCD and chemotherapy, the MCD of 19 patients were studied before and after neo-adjuvant chemotherapy. The clinical characteristics of these 19 patients before chemotherapy were summarized in Table 3. The average ranks of MCD before and after chemotherapy were 3.5 and 7.8 respectively (Table 4); the latter was significantly higher than the former (*P* = 0.002).

**Table 3:** Clinical characteristics of subjects with neo-adjuvant chemotherapy (N = 19)

Age (year)	47.0 (45.0, 53.0)
Left breast cancer	7 (36.8%)
Right breast cancer	12 (63.2%)
Max diameter of cancer (cm)	2.5 (2.0, 4.0)
Infiltrating lobular carcinoma	2 (10.5%)
Infiltrating ductal carcinoma	17 (89.5%)
Axillary lymph node metastasis	5 (26.3%)
ER+	11 (57.9%)
PR+	13 (68.4%)
HER-2+	7 (36.8%)
Ki67	20.0% (10.0%, 60.0%)
Luminal A	4 (21.1%)
Luminal B	4 (21.1%)
HER-2 overexpression	7 (36.7%)
ER-, PR- and HER-2-	4 (21.1%)

Abbreviations: Continuous variables are presented as median (with interquartile range); categorical variables are presented as number (percentage). ER+, estrogen receptor positive; PR+, progesterone receptor positive; HER-2+, human epidermal growth factor receptor-2 positive; Ki67, Ki67 nuclear antigen.

**Table 4:** The difference of mast cell infiltration density before and after neo-adjuvant chemotherapy (N = 19)

Neo-adjuvant chemotherapy	N	Average rank	Z	*P*
Before	19	3.5	-3.078	***0.002***
After	19	7.8		

N, number of subjects in each group; Z, Z-value.

## DISCUSSION

The occurrence, development and prognosis of breast cancer are related to gender, age, tumor size, histological type and grade, axillary lymph node metastasis, female hormone receptors, HER-2 expression and nuclear protein Ki67 [28-30]. While, the role of mast cell in the pathophysiological process of breast cancer is still unclear. In this report, we found that the MCD in patients younger than 50 years old was significantly higher than that in patients age ≥ 50; secondly, the MCDs in ER or PR positive patients were significantly higher than MCDs in ER or PR negative patients; thirdly, the MCD in patients with Ki67 ≤ 14% was also significantly higher than MDC in patients with Ki67 > 14%; fourthly, MCD of patients with invasive ductal carcinoma was significantly higher than MCD of patients with invasive lobular carcinoma; fifthly, no significant distribution difference of MCD was found to be associated with max diameter of cancer, lymph node metastasis and HER-2; finally, further analysis found that MDC was significantly higher in patients after neo-adjuvant chemotherapy.

Age is a key predictor of breast cancer biologic and etiologic heterogeneity and may be independent to menopausal status [31]. Our study showed that the degree of mast cell infiltration is much higher in women younger than 50 years old. Mast cells are more abundant in the lower incidence group of breast cancer, which might suggest that mast cells may have an inhibiting role in the development of breast cancer. The expression profiles of female hormone receptors, ER and PR, was found to be independent prognostic factors for the development, progression and treatment of breast cancer [32], our data showed that mast cell density is higher in the ER and/or PR positive tissue, which suggests that mast cells were inversely associated with negative factors of breast cancer. The Ki67, a nuclear protein that is associated with cellular proliferation, is a cellular marker for tumor proliferation [30]. Our data showed that mast cell infiltration is abundant in cancer tissues with Ki67 expression ≤ 14%. Although we could not conclude that mast cell is an inhibiting factor for cancer cell proliferation, our data showed that mast cell density is also inversely associated with the degree of tumor proliferation, which suggest again that mast cell might has an inhibitory effect on cell proliferation in cancer tissue. Invasive ductal carcinoma and invasive lobular carcinoma are two major histological types of breast cancer [28-30]. Our data showed that MCD of patients with invasive ductal carcinoma was significantly higher than MCD of patients with invasive lobular carcinoma. Although we could not give a satisfactory explanation to this difference, our data suggested that mast cell might play different roles in these two cancer types. Our paired intervention study showed that MDC was significantly higher in patients after neo-adjuvant chemotherapy, this phenomenon could be explained by the inherent role of mast cell in the immune response, that is, chemotherapy may induce tissue damage, and high density of mast cell in tissue after chemotherapy is important for tissue repair. HER-2 is also a key molecule participates in breast cancer and max diameter of cancer and lymph node metastasis were reported to be associated with breast cancer [22, 25]. It is interesting that our data showed that mast cell infiltration is not associated with these factors. Our data disagreed with previous study on the association between mast cell infiltration and lymph node metastasis [33].

According to the different combinations of ER, PR, HER-2 and Ki67 expression, breast cancer is divided for Luminal A type (ER+, PR+ ≥20%, HER-2- and Ki67 ≤ 14%); Luminal B type (ER+ and/or PR+, HER-2- and Ki67 > 14% or HER-2+); HER-2 over expression type (ER-, PR-, HER-2 over-expressed) and triple negative type (ER-, PR-, HER-2-) [27]. The biological characteristics and treatment differed between types. Of Luminal A type, the prognosis is relatively good; the effect of endocrine therapy is good; and do not need chemotherapy generally [27]. Of Luminal B type, the prognosis is second to Luminal A type; the effect of endocrine therapy is a bit poor and chemotherapy is needed. Of HER-2 overexpression and triple negative types, the effect of endocrine therapy is poor, while, the effect of chemotherapy is good [27]. Of triple negative type, treatments were ineffective except chemotherapy; the prognosis is poor and breast cancer is easy to recur and metastasize [27]. Our data showed that the mast cell infiltration is higher in Luminal A or B patients, although this is not a prospective study focused on the associations between mast cell infiltration and clinical progress on breast cancer, higher MCD associated with Luminal A and B types might suggest that mast cell infiltration is a good prognostic indicator.

## MATERIALS AND METHODS

### Participants

The study was approved by the Review Board of Nanjing Drum Tower Hospital and conducted according to the principles of the World Medical Association Declaration of Helsinki. All subjects provided written informed consent prior to participating in this study. The participants were simultaneously informed of their right to repeal consent by them or their kin, caretakers, or guardians.

Needle biopsy or surgical resection samples were collected from Nanjing Drum Tower Hospital and The Traditional Chinese Medicine Hospital of Jiangsu Province between 2014 January to June 2016. All specimens were fixed by 10% neutral formalin within 5 min after biopsy or surgical resection and embedded in paraffin. Breast cancer was diagnosed by histological examination using needle biopsy or surgical resection samples. Invasive carcinoma of no special type was double confirmed by two pathologists. Data of age, tumor size, lymph node metastasis and expression of ER, PR, HER-2 and Ki67 were collected. Totally, 219 females with age range 29 – 83 were recruited in this study. Of the 219 subjects, 19 were treated with neo-adjuvant chemotherapy. Chemotherapy protocol: three cycles of TEC (docetaxel, 75 mg/m2 body surface area; epirubicin, 50 mg/m2 body surface area; and cyclophosphamide, 500 mg/m2 per body surface area) for three weeks. All above clinical data of these 19 patients before and after chemotherapy were recorded.

### Histological and molecular examinations

Following fixation of the samples, 4–5-μm thick slices were stained by hematoxylin and eosin (H&E) according to standard pathological procedures.

For mast cell staining, the sections were deparaffinized and hydrated in distilled water; sections were then stained by toluidine blue in 50 ml solution containing 20 mg toluidine bule, 5 ml 70% alcohol, 45 ml 1 % sodium chloride (pH 2.0 ~ 2.5) for 2-3 minutes; after washing in distilled water three times, the sections were dehydrated quickly through 95% and 2 changes of 100% alcohol; then, slides were cleared in xylene (2 changes, 3 minutes each); coverslip with resinous mounting medium.

For immunohistochemical staining of ER, PR, HER-2 and Ki67; the non-specific antigens were blocked by 10% skimmed milk diluted in phosphate buffered saline (PBS) buffer; after rinsing in PBS three times, the primary antibodies (1: 200 diluted) were added to the section and incubated in 4°C for 2 hours; rinsing the slides in PBS three times; biotinylated secondary antibodies (Biovisualab, Shanghai, China) were added to the sections and incubated in room temperature for 20 minutes; rinsing the slides in PBS three times; incubate sections in ABC-Peroxidase Solution (VECTASTAIN ^®^ ABC kit, Burlingame, CA) for 30 minutes at room temperature; rinse in PBS for 3 x 2 min; incubate sections in peroxidase substrate solution; rinse in PBS-Tween 20 for 3x2 min; rinse in running tap water for 2-5 minutes; dehydrate through 95% ethanol for 1 minute, 100% ethanol for 2x3min; Clear in xylene for 2x5min; coverslip with mounting medium. The primary antibodies against ER, PR, HER-2 and Ki67 were purchased from Sigma-aldrich (SIGMA-ALDRICH, Nanjing, China).

Immunohistochemical results determination of ER and PR was followed the “American Society of Clinical Oncology/College of American Pathologists Guideline Recommendations for Immunohistochemical Testing of Estrogen and Progesterone Receptors in Breast Cancer” [28]. Immunohistochemical results determination of ER and PR was followed the “Recommendations for Human Epidermal Growth Factor Receptor 2 Testing in Breast Cancer: American Society of Clinical Oncology/College of American Pathologists Clinical Practice Guideline Update” [29]. Immunohistochemical results determination of ER and PR was followed the “Assessment of Ki67 in Breast Cancer: Recommendations from the International Ki67 in Breast Cancer Working Group” [30].

Histological examinations and photographs were taken using a NanoZoomer 2.0 RS optical microscope (Hamamatsu, Japan). For each positive slide, we randomly selected the counting area using x100 under the microscope; then we selected 20 counting views randomly using x400; the positive cells of the 20 views were then counted and the average was calculated.

### Statistical analysis

The distribution tendency of all data were evaluated before further statistical analysis, skew distributional variables are presented as median (with interquartile range) or average ranks; categorical variables are presented as number (percentage). Differences among groups were examined using rank test or chi-square test. Paired data were compared by Wilcoxon Signed Rank Sum Test. Difference among three or more groups were determined by Kruskal-Wallis test. The level of statistical significance was set at 0.05. Statistical analyses were performed using SPSS 19.0 for Windows (SPSS, Inc., Chicago, IL).

## CONCLUSION

Our data regarding the correlations between mast cell infiltration and known molecular factors and other issues suggested that mast cell played a beneficial role in inhibiting breast cancer.
